# Genome-Wide Gene Expression Analysis of Mtb-Infected DC Highlights the Rapamycin-Driven Modulation of Regulatory Cytokines *via* the mTOR/GSK-3β Axis

**DOI:** 10.3389/fimmu.2021.649475

**Published:** 2021-04-16

**Authors:** Marilena P. Etna, Martina Severa, Valerio Licursi, Manuela Pardini, Melania Cruciani, Fabiana Rizzo, Elena Giacomini, Gianfranco Macchia, Orazio Palumbo, Raffaella Stallone, Massimo Carella, Mark Livingstone, Rodolfo Negri, Sandra Pellegrini, Eliana M. Coccia

**Affiliations:** ^1^ Department of Infectious Diseases, Istituto Superiore di Sanità, Rome, Italy; ^2^ Department of Biology and Biotechnology, Sapienza University, Rome, Italy; ^3^ Proteomics Core Facility, Istituto Superiore di Sanità, Rome, Italy; ^4^ Division of Medical Genetics, Fondazione IRCCS-Casa Sollievo della Sofferenza, San Giovanni Rotondo, Italy; ^5^ Cytokine Signaling Unit, Inserm, Institut Pasteur, Paris, France

**Keywords:** host-directed therapy, *Mycobacterium tuberculosis*, tuberculosis, rapalogs, IFN, autophagy, transcriptome, translatome

## Abstract

In human primary dendritic cells (DC) rapamycin—an autophagy inducer and protein synthesis inhibitor—overcomes the autophagy block induced by *Mycobacterium tuberculosis* (Mtb) and promotes a Th1 response *via* IL-12 secretion. Here, the immunostimulatory activity of rapamycin in Mtb-infected DC was further investigated by analyzing both transcriptome and translatome gene profiles. Hundreds of differentially expressed genes (DEGs) were identified by transcriptome and translatome analyses of Mtb-infected DC, and some of these genes were found further modulated by rapamycin. The majority of transcriptome-associated DEGs overlapped with those present in the translatome, suggesting that transcriptionally stimulated mRNAs are also actively translated. *In silico* analysis of DEGs revealed significant changes in intracellular cascades related to cytokine production, cytokine-induced signaling and immune response to pathogens. In particular, rapamycin treatment of Mtb-infected DC caused an enrichment of IFN-β, IFN-λ and IFN-stimulated gene transcripts in the polysome-associated RNA fraction. In addition, rapamycin led to an increase of IL-12, IL-23, IL-1β, IL-6, and TNF-α but to a reduction of IL-10. Interestingly, upon silencing or pharmacological inhibition of GSK-3β, the rapamycin-driven modulation of the pro- and anti-inflammatory cytokine balance was lost, indicating that, in Mtb-infected DC, GSK-3β acts as molecular switch for the regulation of the cytokine milieu. In conclusion, our study sheds light on the molecular mechanism by which autophagy induction contributes to DC activation during Mtb infection and points to rapamycin and GSK-3β modulators as promising compounds for host-directed therapy in the control of Mtb infection.

## Introduction

As reported by World Health Organization, millions of people in the world continue to fall ill each year with tuberculosis (TB), one of the top killer infectious disease caused by *Mycobacterium tuberculosis* (Mtb) (1). The reasons behind Mtb capacity to maintain this sad primacy must be sought in the different strategies employed by this pathogen to survive inside the host, ranging from the establishment of a latent infection to several mechanisms for evading the immune response (2). In this context, the host innate immune system and its plethora of defense mechanisms critically influence the fate of the infection (2). Among innate immune cells, macrophages and dendritic cells (DC) are early infected by Mtb and, in turn, these cells orchestrate the immune response against the pathogen. In particular, DC, as professional antigen presenting cells (APC), undergo phenotypic modifications and produce a panel of pro-inflammatory and regulatory cytokines which tune the immune response by acting on different cell populations, including naïve T cells (3). A critical prerequisite for DC activation is the sensing of Mtb-associated molecular structures by pattern recognition receptors whose stimulation leads to a range of cellular events that contribute to host mycobacterial control. However, during its co-evolution with the host, Mtb has evolved numerous evasion mechanisms to hijack innate immune response, such as cytosolic escape, block of phagosome maturation, apoptosis, inflammasome activation/modulation as well as autophagy inhibition (4). In particular, the relevance of autophagy in the anti-mycobacterial response is well documented. Indeed, it was shown that, in order to promote its own survival, virulent Mtb impairs phagosome maturation by altering the acidic, hydrolytic environment of the intracellular compartment (5), it compromises the autophagy-mediated antigen-processing capacity of macrophages and DC (6, 7) and blocks autophagosome formation and maturation (2, 8, 9). Interestingly, in the last decade many authors have investigated the effect of autophagy modulators to improve anti-mycobacterial host cell functions and proposed their use as potential therapeutic treatment. Since autophagy is regulated by several intracellular pathways and some of them converge on the master regulatory kinase mammalian target of rapamycin (mTOR), autophagy-targeting strategies can either inhibit mTOR or can be mTOR-independent (10). In this context, we previously showed that the block of autophagy caused by Mtb in human DC is overcome by rapamycin, an autophagy inducer and mTOR inhibitor. Moreover, treatment with rapamycin leads also to increased expression of interferon-β (IFN-β) and increased production of the pro-Th1 cytokine, interleukin 12 (IL-12) (9).

In the current study, we investigated the molecular mechanisms underlying rapamycin-mediated immune-stimulatory action, by studying both transcriptome and translatome gene profiles of DC infected with Mtb in presence or absence of rapamycin. We found that, during Mtb infection, the drug can act as a selective inducer of protein translation, by promoting the association of type I and type III IFN mRNAs to polysomes and by preventing the inhibition of the constitutively active glycogen synthase kinase 3 beta (GSK-3β) through which rapamycin impinges on the balance of secreted pro- and anti-inflammatory cytokines. 

## Materials and Methods

### Ethics Statement

Istituto Superiore di Sanità Review Board approved the present research project (CE/13/387). No informed consent was given since the data were analyzed anonymously. Peripheral blood mononuclear cells were isolated from freshly collected buffy coats obtained from healthy voluntary blood donors (Blood Bank of University “La Sapienza”, Rome, Italy).

### DC Preparation and Cell Culture

DC were prepared as previously described (11). Briefly, DC were generated by culturing CD14^+^ monocytes with 50 ng/ml GM-CSF and 1000 U/ml IL-4 (R&D Systems, Minneapolis, MN, USA) for 5 days at 0.5x10^6^ cells/ml in RPMI 1640 (Lonza, Basel, Switzerland), supplemented with 2 mM L-glutamine and 15% Fetal Bovine Serum (FBS) (Lonza). At day 5 the cells were tested for their differentiation status by evaluating CD1a expression (>90% CD1a^+^) and lack of CD14 (>95% CD14^-^). Before infection, the medium was replaced with RPMI without antibiotics and supplemented with 2 mM L-glutamine and 15% FBS. Cytokine deprivation did not affect DC survival rate, which was >90%.

### Antibodies (Abs) and Other Reagents

For immunoblotting analysis, rabbit anti-phospho-p70S6K (S371), rabbit anti-phospho- p70S6K (T389), rabbit anti-GSK-3β, rabbit anti-phospho-GSK-3β (S9), rabbit anti-phospho-GS (S641), rabbit-anti-p38, rabbit-anti-phospho-p44/42 (T202/Y204), rabbit anti-phospho-p38 (T180/Y182) (Cell Signaling, Danvers, MA, USA), mouse anti-actin (Sigma-Aldrich, St. Louis, MO, USA), and horseradish peroxidase-conjugate secondary anti-mouse and anti-rabbit Abs (Santa Cruz Biotechnology) were used. Rapamycin (0.2 µM, Sigma-Aldrich) or Torin 1 (0.5 µM Tocris, Bristol, UK) were added to DC culture 4 h after Mtb-infection for studying mammalian target of rapamycin complex 1 (mTORC1) and mTOR inhibition respectively. For the selective inhibition of GSK-3β and p70S6K1 respectively, SB216763 (5 µM, Sigma-Aldrich) and PF4708671 (0.1 µM, Sigma-Aldrich) were used to treat DC 30 min before infection and rapamycin treatment. To evaluate rapamycin bystander effect, DC were treated for 30 min before infection and rapamycin treatment with SB203580 (10 µM, Sigma-Aldrich) or SB202129 (10 µM, Sigma-Aldrich) to inhibit p38 or with PD980509 (0.1 µM, Sigma-Aldrich) to block p44/42. 

### Bacterial Strain Description and Preparation

Mtb H37Rv (ATCC 27294; American Type Culture Collection) was grown as previously described (8). Logarithmically growing cultures were centrifuged at 800 rpm for 10 min to eliminate clumped mycobacteria and then washed three times in RPMI 1640. Mycobacteria were re-suspended in RPMI 1640 containing 10% FBS and then stored at - 80°C. Bacterial frozen vials were thawed and bacterial viability was determined by counting the number of colony forming units. All bacterium preparations were tested for endotoxin contamination (<1 Endotoxin Unit/ml) by the Limulus lysate assay (Lonza). DC cultures were then infected with a multiplicity of infection (MOI) of 1 bacterium/cell as previously described (8).

### Total RNA Extraction, Quantification, and Quality Assessment

Total RNA was isolated from 10x10^6^ infected or not infected DC, treated or not with rapamycin using Trizol (Invitrogen, Thermo Fisher Scientific) and by following manufacturer’s recommendations. RNA was quantified using a Nanodrop 2000 spectrophotometer (Thermo Fisher Scientific, Waltham, MA, USA) and quality assessed with an established cut-off of 1.8 for both the 260/280 and 260/270 absorbance ratios. RNA integrity was instead inspected by bioanalyzer analysis (Agilent Technologies, Santa Clara, CA, USA) considering a cut-off of 1.8 for 28S/18S ratio.

### Isolation of Polysome-Associated RNA

For RNA fraction isolation, 10×10^6^ cells for untreated DC (CTRL), DC treated for 12 h with rapamycin (0.2 µM) and DC infected for 16 h with Mtb (MOI 1) and treated or not with rapamycin (12 h, 0.2 µM) were used. Polysome-associated RNA were prepared as previously described (8). Briefly, each gradient was fractionated in BSL3 facility by hand collection from the top of the gradient into 2 samples, light phase (low polysome occupancy) and heavy phase (high polysome occupancy). The hand collection method was previously tested to parallel with fraction collection using a fractionator coupled with an UV optical reader (A 254nm). The obtained fractions were immediately mixed with an equal volume of Trizol (Invitrogen, Thermo Fisher Scientific) for later RNA isolation following Trizol manufacturing instructions.

### Microarray Analysis

Gene expression profiling was performed using the GeneChip^®^ Human Transcriptome Array 2.0 (Thermo Fisher Scientific, Waltham, MA, USA) including more than 6.0 million distinct probes covering both coding and non-coding genes built on transcript mappings from hg19 human reference sequence (GRCh37). A total of 400,000 full length transcripts were combined from available public data sources: RefSeq NCBI, UCSC Genes, Vega, GENCODE, Ensembl, Mammalian Gene Collection. Additional long non-coding content was combined from the UCSC genome browser, noncode.org, Broad Human Body Map. Probes are distributed across the full length of the gene including specific probes (>339.000 probe sets) covering splice junction, providing a complete and accurate picture of overall gene expression with the additional ability for transcript isoform analysis.

For each sample enrolled for the study, 100 ng of total RNA were processed according to the GeneChip Whole Transcript Sense Target Labeling Assay following the manufacturer’s instructions (Thermo Fisher Scientific, Waltham, MA, USA). Briefly, a random priming method was used to generate cDNA from all RNA transcripts present in a sample. The random primers incorporate a T7 promoter sequence, which is subsequently used in an *in vitro* transcription to produce antisense cRNA fragments. Single stranded cDNA complementary to the cRNA is then produced, in the sense orientation, where a modified dUTP is incorporated instead of dTTP. The modified dUTP is subsequently recognized by the enzymes Uracil-DNA glycosylase and human apurinic/apyrimidinic endonuclease 1 which will cut the DNA, resulting in fragmentation of the cDNA. Each DNA fragment is end-labelled with biotin using terminal deoxynucleotidyltransferase before being hybridized to the arrays for 16 h at 45°C in a GeneChip Hybridization Oven 640. Following hybridization and post hybridization washes each array were scanned using the GeneChip Scanner 3000 7G to generate the raw data (.CEL file). The QC steps of the experiment were performed using Expression Console v1.4 (Thermo Fisher Scientific, Waltham, MA, USA) software.

CEL files were first processed with the Affymetrix collection of command line programs Analysis Power Tools (APT, v1.16.0) using the *apt-probeset-summarize* command in order to estimate the intensity at gene level with the *RMA-sketch* normalization. In this step background corrected raw data were Log2-transformed and quantile-normalized following the Robust Multichip Average (RMA) procedure as described in Irizarry et al. (12). For gene annotation the file “HTA-2_0.na34.hg19.transcript” (obtained from Affymetrix) was used. R v3.1.2 (www.r-project.org) environment and Bioconductor (www.bioconductor.org) packages were used for the following preprocessing and statistical analysis. PCA was performed with the native R function *prcomp* on RMA-normalized data and the first two principal components were plotted with *ggplot2* package functions. Hierarchical clustering of sample Euclidean distances was performed with *hclust* and *dist* native R functions and the resulting heatmap was plotted with *gplots* package *heatmap.2* function. Finally, *tRanslatome* package pipeline was used to find out the probe sets that showed significant (FDR < 0.01) differential expression between experimental conditions and to perform the Gene Ontology (GO) Database (13) enrichment analysis, internally using the *limma* - Linear Models for Microarray Data - (14) and *topGO* methods, respectively. The GO and pathway enrichment analysis were obtained using *clusterProfiler* (15) package with annotation of KEGG (16) and Reactome (17) databases.

Microarray data accompanying this paper are available through NCBI Gene Expression Omnibus (GEO) repository, under accession number GSE163531.

### Quantitative Real-Time PCR and Digital PCR

For the analysis of IL12p40 and IL-10 mRNA levels, total RNA was reverse transcribed as previously described (11) and then analyzed using the appropriate TaqMan assay (Applied Biosystems) and TaqMan Universal Master Mix II (Applied Biosystems) according to the manufacturer’s instructions. Transcripts expression were normalized to the GAPDH level by using the equation 2^-ΔCt^; the values are mean ± SEM of triplicate determinations.

The validation of translatome data was conducted by Digital PCR on total and heavy phase-associated RNA that was reverse transcribed as previously described (8). In particular, experiments were carried out to test the mRNA copy number of *IFNA1*, *IFNB1*, *IFNL1* and some IFN stimulated genes (ISGs). Among ISGs we tested CXCL9, CXCL11, two chemokines involved in T cell recruitment; IFN induced with helicase C domain 1 (IFIH1, alias MDA5) - an RNA sensing molecule recently proposed as innate restriction factor for Mtb growth (18), IFN induced protein with tetratricopeptide repeats (IFIT3) - the bridging adapter of TANK binding kinase 1 (TBK1)/mitochondrial antiviral signaling protein (MAVS) implicated in TBK1 activation and IFN regulatory factor 3 (IRF3) phosphorylation (19) - and IRF7 - a transcription factor stimulated by the autocrine action of IFN-β induced in STING/IRF3-dependent manner (20). Digital PCR experiments were carried out on the BioMark HD System (Fluidigm, San Francisco, CA, USA) by loading cDNA samples into Fluidigm’s 37K Digital Array microfluidic chip as previously described (8). Briefly, Fluidigm’s 37K Digital Array consists of 48 panels, each of which is further partitioned into 770 reaction chambers. The reaction for each panel was set up with the specific TaqMan assay probe and with TaqMan Universal Master Mix II (Applied Biosystems) into a final volume of 5 μl. For each sample, six serial dilutions were loaded in triplicate reactions and the chip was then thermocycled and imaged on Fluidigm’s BioMark HD real-time PCR system. Positive chambers that originally contained 1 or more molecules has been counted by the Digital PCR analysis software (Fluidigm) and only templates that yielded 150–360 amplified molecules per panel were chosen for technical replication in order to obtain absolute quantification of target RNA copy number.

### DC Transfection and Gene Silencing by siRNA

Predesigned siRNA oligonucleotide for GSK-3β (iGSK-3β) was obtained from Ambion (Thermo Fisher Scientific). A negative-control siRNA oligonucleotide [neg ctrl (Ambion, Thermo Fisher Scientific)] was used to address the specificity of the observed effect to the specific GSK-3β sequence. SiRNA transfection efficiency was determined as previously described (8). For each condition, 4x10^5^ DC were plated in a 12-well plate. Lipoplexes were prepared in Opti-MEM serum-free medium (Invitrogen, Thermo Fisher Scientific) at 1:1 ratio. Briefly, siRNA plus fluorescent oligonucleotide and 10 μl of Lipofectamine (1 mg/mL) were diluted separately in 250 μl of Opti-MEM medium.

The Lipofectamine solution was added to the siRNA solution and placed for 5 min at room temperature and then added to DC. Four hours (h) after incubation, medium was replaced with fresh Opti-MEM medium supplemented with 15% FCS and 2 mM L-glutamine for at least 8 h. After the indicated time, cells were infected with Mtb and, where required, treated with rapamycin. After the indicated time, DC were harvested to recover culture supernatants and to isolate RNA. Transfection effect on DC viability, maturation and activation was evaluated as previously described (8).

### Immunoblotting Analysis

Total protein extracts were prepared from 1x10^6^ infected or not infected DC, treated or not with rapamycin by using the CelLytic cell lysis reagent (Sigma-Aldrich) supplemented with protease and phosphatase inhibitor cocktails and by following manufacturer’s recommendations.

For P-p70S6K S371, P-p70S6K T389, total GSK-3β, P-GSK-3β S9, P-GS S641, P-p44/42 T202/Y204, total p38 and P-p38 Y180/Y182 determination 25 μg of total protein extract were separated on 10% NuPAGE Bis-Tris gel (Invitrogen, Thermo Fisher Scientific) and electroblotted onto nitrocellulose membranes (GE Healthcare, Pittsburgh, PA, USA). Blots were incubated with primary Abs in 5% nonfat dry milk in TBS plus 0.1% Tween20 (Sigma-Aldrich) overnight at 4°C. Detection was achieved using horseradish peroxidase-conjugate anti-rabbit or anti-mouse or anti-goat (Santa Cruz) secondary Abs and visualized with Clarity Western ECL Substrate (Bio-Rad, Segrate, Italy). The quantifications of P-p70S6K S371, P-p70S6K T389, total GSK-3β, P-GSK-3β S9, P-GS S641, P-p44/42 T202/Y204, total p38 and P-p38 Y180/Y182 were performed by calculating their ratio compared to the actin level by using ImageLab software (Bio-Rad) and then calculating the ratio among infected/treated vs uninfected conditions.

### Cytokine Determination

Supernatants of DC cultures were harvested 24 h after infection, filtered (0.2 μm) and stored at −80°C. The production of IL-1β, TNF-α, IL-6, IL-12, and IL-10 was measured by human Inflammatory Cytokine bead array kit (BD Bioscience). IL-23 release was instead assayed by ELISA (R&D Systems).

### Statistical Analysis

Statistical analysis was performed using One-way Repeated-Measures ANOVA. In case of significant ANOVA, the pairwise comparisons were carried out by the use of the post-hoc Tukey’s test, in order to test the significance of the difference between two stimulation effects. In all the cases above, a p value < 0.05 was considered statistically significant: “*” refers to the p-value ≥ 0.01 and < 0.05; “**” refers to the p-value is ≥ 0.001 and < 0.01; “***” the p-value is <0.001. Data analysis was carried out using native functions of R language version 4.0.3.

## Results

### Profiling of Human Primary DC Infected With Mtb and Treated With Rapamycin

We have previously shown that in human primary DC the autophagy inducer rapamycin overcomes the block exerted by Mtb on autophagosome/lysosome fusion, and enhances IL-12 secretion driving a protective Th1 response (9). Here, to further investigate how rapamycin, known to inhibit translation by blocking mTORC1, impacts on the immunoregulatory properties of Mtb-infected DC, a comparative analysis of global transcriptome and translatome profiles was conducted (**Supplementary Figure 1**).

**Figure 1:**
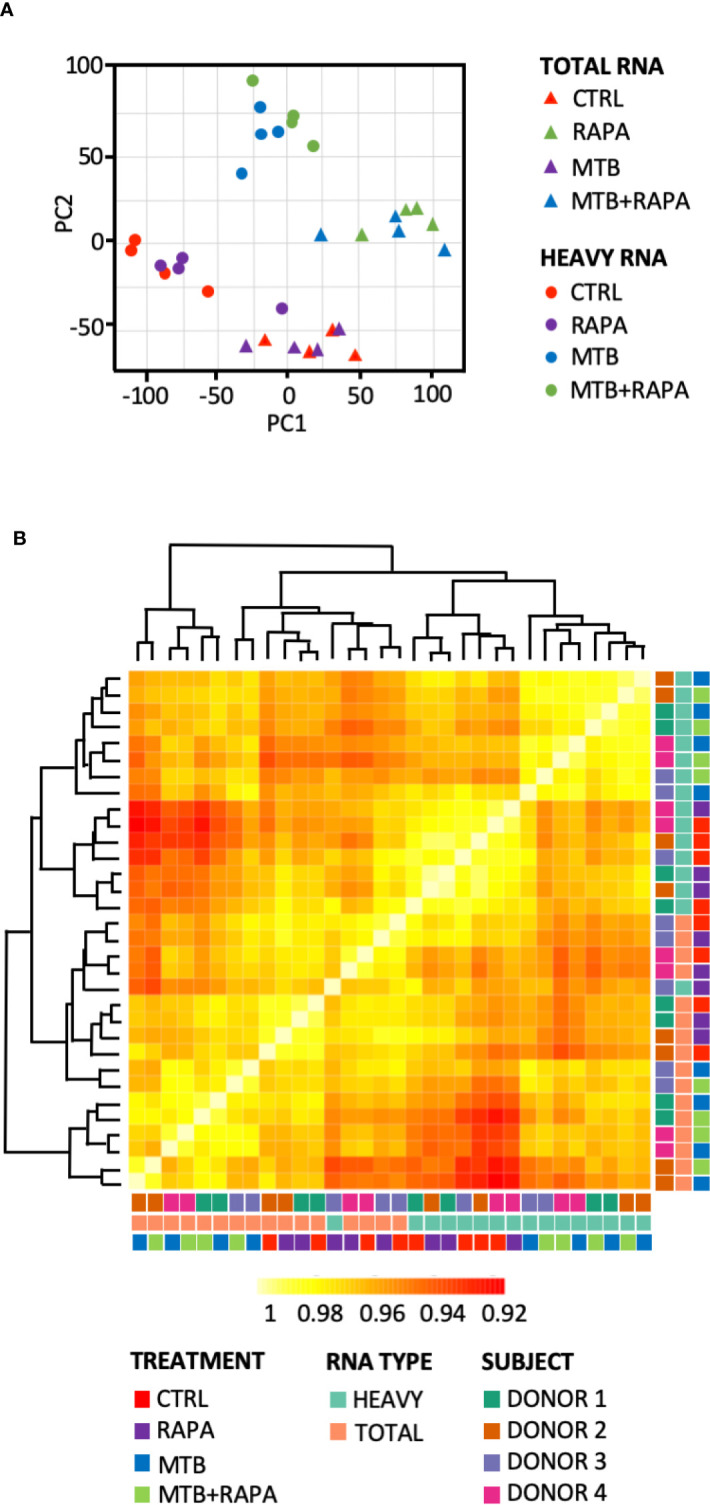
Analysis of transcriptome and translatome of human DC infected with Mtb and treated with rapamycin. DC isolated from 4 healthy donors were left unstimulated (CTRL), treated with rapamycin (RAPA) or infected with Mtb alone (MTB) or in presence of rapamycin added 4 h post infection (MTB+RAPA). Cells were harvested 16 h post infection for total and polysome-associated RNA isolation and microarray analysis. **(A)** Principal component analysis of gene expression profile of samples. Triangles indicate total RNA samples (TOTAL), circles refer to RNA fraction associated with polysome chains (HEAVY). **(B)** Heatmap showing hierarchical clustering of sample correlation. The dendrogram clusters together the significantly regulated treatment conditions with most similar expression profiles among the 4 donors. Correlation is represented by a color code according to the legend where yellow indicates higher correlation. A color code was used to distinguish among treatment conditions (CTRL, RAPA; MTB, MTB+RAPA), RNA type (TOTAL vs HEAVY) and DC culture prepared from different subjects (D1, D2, D3, D4).

Total and polysome-associated (heavy) RNA samples were prepared from uninfected DC or DC infected for 16 h with live H37Rv Mtb alone or in combination with rapamycin. Principal component analysis and sample correlation/clustering study of our gene expression profiles clearly showed a distinct pattern of total and heavy RNA samples and of Mtb-infected *vs* uninfected DC for the four analyzed donors (**Figure 1**). In particular, the MA-plots displaying the logged intensity ratio (M) *vs* the mean logged intensities (A) of mRNAs whose profiles were significantly modulated in treated and/or infected DC *vs* uninfected cells (adjusted p-value [FDR] < 0.01 and at least 1.5-log2 fold change), revealed 228 and 404 differentially expressed genes (DEGs) in transcriptome and translatome of Mtb-infected DC, respectively. No difference was observed in uninfected cells treated with rapamycin *vs* control cells. When rapamycin was added to Mtb-infected DC, DEGs increased to 378 in the transcriptome and 729 in the translatome (**Supplementary Figure 2**). Interestingly, the majority of genes identified in the transcriptome were found in the list of translatome-modulated genes, suggesting that host mRNAs induced by Mtb alone or in combination with rapamycin are efficiently loaded on polysomes, where actively translated mRNAs reside.

**Figure 2:**
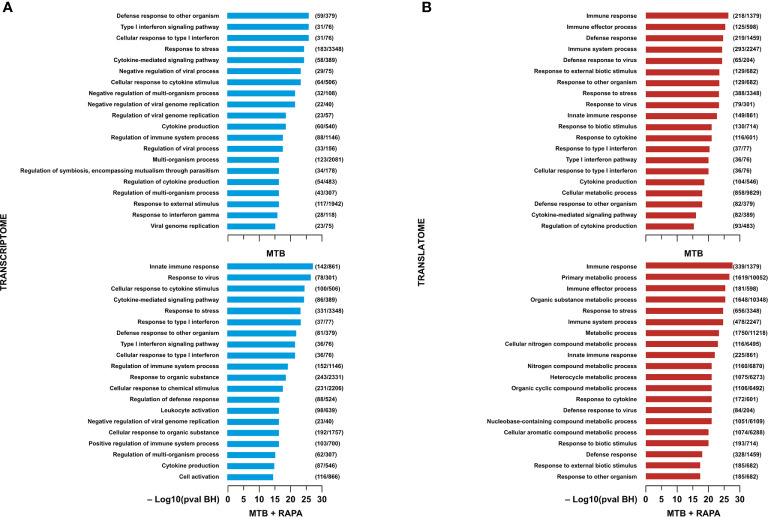
Gene ontology analysis of genes regulated/modulated in transcriptome and in translatome of human DC infected with Mtb alone or in presence of rapamycin. Top 20 gene ontology (GO) terms within the biological process (BP) branch emerged from the list of genes found de-regulated (log2 FC > 1.5; FDR < 0.01) in transcriptome **(A)** (blue panels) and translatome **(B)** (red panels) of human DC infected with Mtb (MTB) and treated with rapamycin (MTB+RAPA). Relevant BP have been graphed by using the formula -log_10_ of their Benjamini-Hochberg false discovery rate adjusted p-values. The numbers next to BP name, represent the total number of genes that may be significantly involved in the corresponding biological processes. The graph displays the classification term enrichment status and term hierarchy.

**Figure 3:**
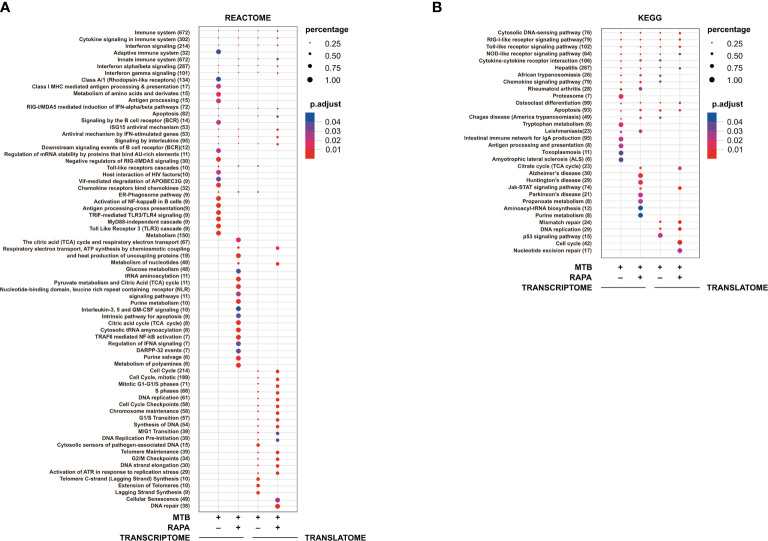
Pathway enrichment analysis of genes modulated in transcriptome and in translatome of human DC infected with Mtb alone or in presence of rapamycin. Pathway enrichment analysis performed using Reactome **(A)** and KEGG **(B)** databases on the list of genes found de-regulated (log2 FC > 1.5; FDR < 0.01) in transcriptome and translatome of human DC infected with Mtb and treated with rapamycin. Dot size is correlated with the ratio between the total number of differentially expressed genes and the number of genes that belong to a pathway. Dots are colored according to the Benjamini-Hochberg false discovery rate adjusted *p*-values (p adjust) from blue (higher *p*-value) to red (lower *p*-value).

Functional GO annotation study and pathway enrichment analysis were performed on the transcriptome- or translatome-associated DEGs and revealed biological processes and signaling cascades associated to Mtb infection and rapamycin treatment (**Figures 2**,**3**). In particular, in the transcriptomes of both Mtb-infected DC and Mtb-infected DC treated with rapamycin there was an enrichment in GO terms related to *cytokine production*, *cytokine signaling* and *innate immune response* (**Figure 2A**). In the GO enrichment profile of the translatome, in addition to *innate immune response*, biological processes linked to *type I interferon (IFN) signaling* and *defense response to other organisms* were found (**Figure 2B**).

Pathway enrichment analysis was performed with both Reactome and KEGG databases and, in line with GO study results, the modulation of signaling cascades related to *antigen processing and presentation*, *TLR receptors signaling*, *cytosolic nucleic acid sensors* and *cytokine-cytokine receptors* was highlighted at transcriptome level for Mtb-infected DC in a rapamycin-independent manner (**Figure 3**). However, while the transcriptome profile of Mtb-infected DC was enriched for the *TRIF-mediated TLR signaling pathway*, mostly involved in CREB-binding protein (CBP)/p300 nuclear translocation for anti-inflammatory cytokine production, the profile of infected DC treated with rapamycin pointed to the *TRAF6-mediated NF-kB activation*, notably involved in the transcription of regulatory and pro-inflammatory cytokines. Interestingly, the analysis of signaling cascades enriched in the translatome of Mtb-infected DC and more pronounced in the presence of rapamycin, revealed modulation of *IFN signaling* and *antiviral mechanism by IFN-stimulated genes*. The addition of rapamycin to infected DC impacted, as expected, on different metabolic pathways, and on *JAK/STAT signaling* at both transcriptome and translatome levels.

### Rapamycin Promotes the Association of type I and III IFNs and ISG mRNAs With Polysomes

*In silico* analysis of translatome-associated DEGs indicated that rapamycin up-regulated anti-microbial factors. Among the top genes up-regulated in the translatome of Mtb-infected DC alone or in presence of rapamycin, type I and III IFNs and several ISGs encoding for chemokines, transcription factors, innate immune receptors and antimicrobial guanylate binding proteins were found. All the above proteins are involved in host protection against microbial injuries (**Table 1**).

**Table 1:** List of top genes de-regulated at the translatome level in human DC after Mtb infection and rapamycin treatment.

GENES UP-REGULATED BY RAPAMYCIN IN MTB-INFECTED DC AT TRANSLATOME LEVEL					
Gene ID	Gene Name	Translatome log2 FC		Transcriptome log2 FC	
		MTB	MTB+RAPA	MTB	MTB+RAPA
NM_005409	**CXCL11**	6.28	7.54	5.61	5.40
NM_052941	**GBP4**	5.31	6.14	4.76	5.06
NM_0011300	**CCL20**	4.69	5.73	3.64	4.77
NM_001838	**CCR7**	4.20	5.31	3.38	4.02
NM_001547	**IFIT2**	4.91	5.28	4.57	4.44
NM_002416	**CXCL9**	4.32	5.04	3.73	3.75
NM_001031	**IFIT3**	4.47	4.72	4.33	4.11
NM_001134	**GBP5**	3.51	4.31	3.48	4.19
NM_002981	**CCL1**	2.60	4.29		4.20
NM_022168	**IFIH1**	3.63	4.24	3.60	3.59
NM_000856	**GUCY1A3**		2.97		1.71
NM_004120	**GBP2**	2.05	2.86	1.81	2.55
NM_002176	**IFNB1**	1.78	2.75		1.84
NM_172140	**IFNL1**	1.59	2.35	1.50	1.65
NM_000857	**GUCY1B3**		2.04		
NM_004031	**IRF7**		1.69		
NM_024013	**IFNA1**		1.53		
**GENES DOWN-REGULATED BY RAPAMYCIN IN MTB-INFECTED DC AT TRANSLATOME LEVEL**					
**Gene ID**	**Gene Name**	**Translatome log2 FC**		**Transcriptome log2 FC**	
		**MTB**	**MTB+RAPA**	**MTB**	**MTB+RAPA**
NM_003246	**THBS1**	3.67	2.94	3.02	2.95
NM_0011357	**TP53INP1**	-2.23	-1.50		
NM_001005	**TRIM37**		-1.62		
NM_003544	**HIST1H4B**		-1.94		
NM_006068	**TLR6**	-2.39	-2.86	-1.50	
NM_001237	**CCNA2**		-3.26		
NM_003537	**HIST1H3B**		-4.09		

List of top genes showing a statistically significant modulation at translatome level in Mtb-infected DC in presence or absence of rapamycin, with respect to uninfected DC.

FC, fold change; MTB, Mtb-infected DC; MTB+RAPA, Mtb-infected DC treated with rapamycin; CXCL11, C-X-C motif chemokine 11; GBP4, Guanylate Binding protein 4; CCL20, Chemokine (C-C motif) ligand 20; CCR7, C-C Chemokine receptor type 7; IFIT2, Interferon Induced Protein With Tetratricopeptide Repeats 2; CXCL9, C-X-C Motif Chemokine Ligand 9; IFIT3, Interferon Induced Protein With Tetratricopeptide Repeats 3; GBP4, Guanylate Binding protein 5; CCL1, Chemokine (C-C motif) ligand 1; IFIH1, Interferon Induced With Helicase C Domain 1; GUCY1A3, Guanylate cyclase soluble subunit alpha-3; GBP2, Guanylate Binding protein 2; IFNB1, Interferon beta; IFNL1, Interferon lambda 1; GUCY1B3, Guanylate cyclase soluble subunit beta-3; IRF7, Interferon regulatory factor 7; IFNA1, Interferon alfa 1; THBS1, Thrombospondin 1; TP53INP1, Tumor Protein P53 Inducible Nuclear Protein 1; TRIM37, Tripartite Motif Containing 37; HIST1H4B, H4 Clustered Histone 2; TLR6, Toll-like receptor 6; CCNA2, Cyclin A2; HIST1H3B, H3 Clustered Histone 2.

**Figure 4:**
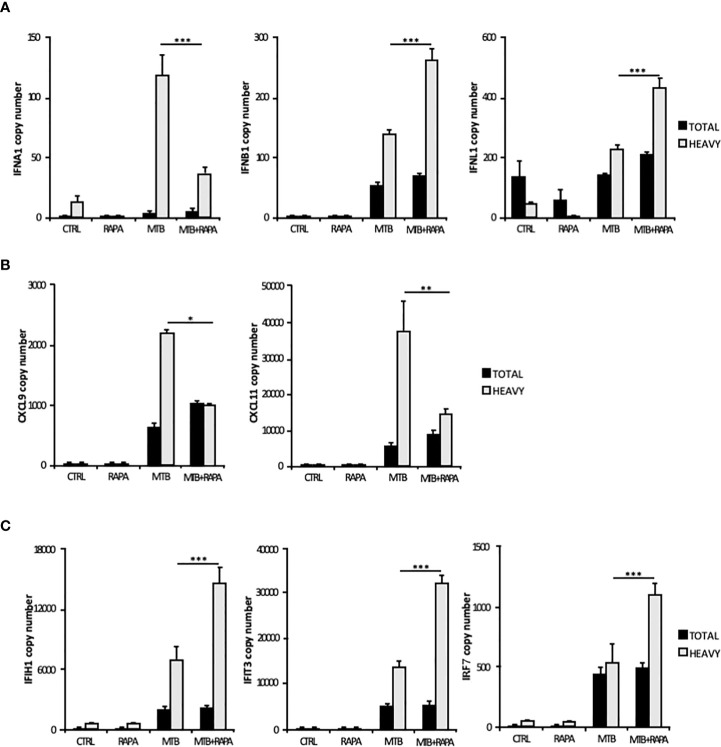
Validation of translatome data on IFNs and IFN-induced gene expression. Total RNA (Total) and high occupancy (Heavy) polysome-associated mRNAs were extracted from untreated DC (CTRL), DC stimulated with rapamycin for 12 h (RAPA) or DC infected for 16 h with Mtb alone (MTB) or in combination with rapamycin (MTB+RAPA, added 4 h after infection). **(A)** *IFNA1*, *IFNB1* and *IFNL1*, **(B)** *CXCL9* and *CXCL11*, **(C)** *IFIH1*, *IFIT3* and *IRF7* copy numbers were determined by digital PCR. Data are represented as the mean copy number per sample ± standard error of 4 experiments performed with RNAs derived from a set of experiments independent than those used in transcriptome/translatome studies and that yielded similar results. Significance was calculated by analysis of variance (ANOVA) followed by multiple comparison performed with Tukey’s test as specified in *Materials and Methods* section.

Considering that the majority of translatome DEGs were also modulated at the transcriptome level (**Table 2**), to identify mRNAs influenced by rapamycin at the translational level, we determined by quantitative digital PCR the copy number of relevant top genes in total RNA and polysome-associated RNA fraction (heavy) (**Figure 4**). We found higher levels of *IFNB1* and *IFNL1* mRNA copies associated to polysomes in Mtb-infected DC in presence of rapamycin, in accordance with the microarray data (**Figures 3A** and **4A**). Some discrepancies with microarray data were instead observed in the number of *IFNA1, CXCL9* and *CXCL11* mRNA copies detected in the polysome heavy fraction that were reduced upon rapamycin treatment of Mtb-infected DC (**Figures 4A, B**). Yet, rapamycin weakly and not significantly reduced the level of CXCL9 and CXCL11 (**Figure 4B** and **Supplementary Figure 3**).

**Table 2:** List of top genes de-regulated at the transcriptome level in human DC after Mtb infection and rapamycin treatment.

GENES UP-REGULATED BY RAPAMYCIN IN MTB-INFECTED DC AT TRANSCRIPTOME LEVEL					
Gene ID	Gene Name	Transcriptome log2FC		Traslatome log2 FC	
		MTB	MTB+RAPA	MTB	MTB+RAPA
NM_002187	**IL12B**	3.78	5.17	4.56	5.82
NM_0011300	**CCL20**	3.64	4.77	4.69	5.73
NM_000417	**IL2RA**	3.79	4.50	4.24	4.88
NM_002981	**CCL1**		4.20	2.60	4.29
NM_000584	**IL8**	3.37	4.07	3.78	4.53
NM_000575	**IL1A**		4.03	3.63	3.95
NM_001838	**CCR7**	3.38	4.02	4.20	5.31
NM_016584	**IL23A**	1.46	3.82	1.94	4.28
NM_001570	**IRAK2**	2.40	3.09	2.81	3.42
NM_003821	**RIPK2**	2.31	3.03	2.18	2.94
NM_001165	**BIRC3**	2.31	2.95	2.47	3.27
NM_021127	**PMAIP1**	1.99	2.64	2.16	3.06
NM_000576	**IL1B**		2.53		2.39
NM_002176	**IFNB1**		1.84	1.78	2.75
NM_001244	**MAP3K8**		1.82	2.60	1.93
NM_0011273	**CFLAR**		1.64		1.66
NM_138723	**BCL2L14**		1.65		2.03
**GENES DOWN-REGULATED BY RAPAMYCIN IN MTB-INFECTED DC AT TRANSCRIPTOME LEVEL**					
**Gene ID**	**Gene Name**	**Transcriptome log2 FC**		**Translatome log2 FC**	
		**MTB**	**MTB+RAPA**	**MTB**	**MTB+RAPA**
NM_001565	**CXCL10**	7.71	6.92	7.44	7.70
NM_005623	**CCL8**	5.19	4.57	4.38	3.96
NM_001548	**IFIT1**	4.65	3.92	4.24	4.14
NM_017414	**USP18**	4.32	3.82	4.40	4.44
ENST000004	**USP41**	3.74	3.32	3.87	3.89
NM_001080	**LILRB1**	1.50		1.77	
NM_006877	**GMPR**	3.65	3.04	3.50	3.59
NM_0011396	**ATF5**	1.70		1.54	
NM_002168	**IDH2**		-1.58		-1.64
NM_003839	**TNFRSF11A**		-1.74		-1.40
NM_000962	**PTGS1**	-1.57	-2.09	-1.53	-2.17
NM_0011280	**PAK1**		-2.09	-1.95	-2.87
NM_004536	**NAIP**		-2.16	-2.05	-3.52

List of top genes showing a statistically significant modulation at transcriptome level in Mtb-infected DC in presence or absence of rapamycin, with respect to uninfected DC.

FC, fold change; MTB, Mtb-infected DC; MTB+RAPA, Mtb-infected DC treated with rapamycin; IL12B, Interleukin 12B; CCL20, Chemokine (C-C motif) ligand 20; IL2RA, Interleukin 2 Receptor Subunit Alpha; CCL1, C-C Motif Chemokine Ligand 1; IL8, Interleukin 8; IL1A, Interleukin 1 Alpha; CCR7; C-C Motif Chemokine Receptor 7; IL23A, Interleukin 23A; IRAK2, Interleukin 1 Receptor Associated Kinase 2; RIPK2, Receptor Interacting Serine/Threonine Kinase 2; BIRC3, Baculoviral IAP Repeat Containing 3; PMAIP1, PMA-Induced Protein 1; IL1B, Interleukin 1B; IFNB1, Interferon beta; MAP3K8, Mitogen-Activated Protein Kinase 8; CFLAR, CASP8 And FADD Like Apoptosis Regulator; BCL2L14, BCL2 Like 14; CXCL10, C-X-C Motif Chemokine Ligand 11; CCL8, C-C Motif Chemokine Ligand 8; IFIT1, Interferon Induced Protein With Tetratricopeptide Repeats 1; USP18, Ubiquitin Specific Peptidase 18; USP41, Ubiquitin Specific Peptidase 41; LILRB1, Leukocyte Immunoglobulin Like Receptor B1; GMPR, Guanosine Monophosphate Reductase; ATF5, Activating Transcription Factor 5; IDH2, Isocitrate Dehydrogenase (NADP(+)) 2; TNFRSF11A, TNF Receptor Superfamily Member 11A; PTGS1, Prostaglandin-Endoperoxide Synthase 1; PAK1, P21 (RAC1) Activated Kinase 1; NAIP, NLR Family Apoptosis Inhibitory Protein.

Among the ISGs whose expression was promoted by rapamycin during Mtb infection, *IFIH1*, *IFIT3* and *IRF7* were validated by digital PCR (**Figure 4C**). As expected, the levels of *IFIH1*, *IFIT3* and *IRF7* mRNAs were induced by Mtb and further enhanced by rapamycin in polysome-associated RNA fraction only.

### The Mtb-Driven Pro-Inflammatory/Regulatory DC Phenotype Is Enhanced by Rapamycin

**Figure 5:**
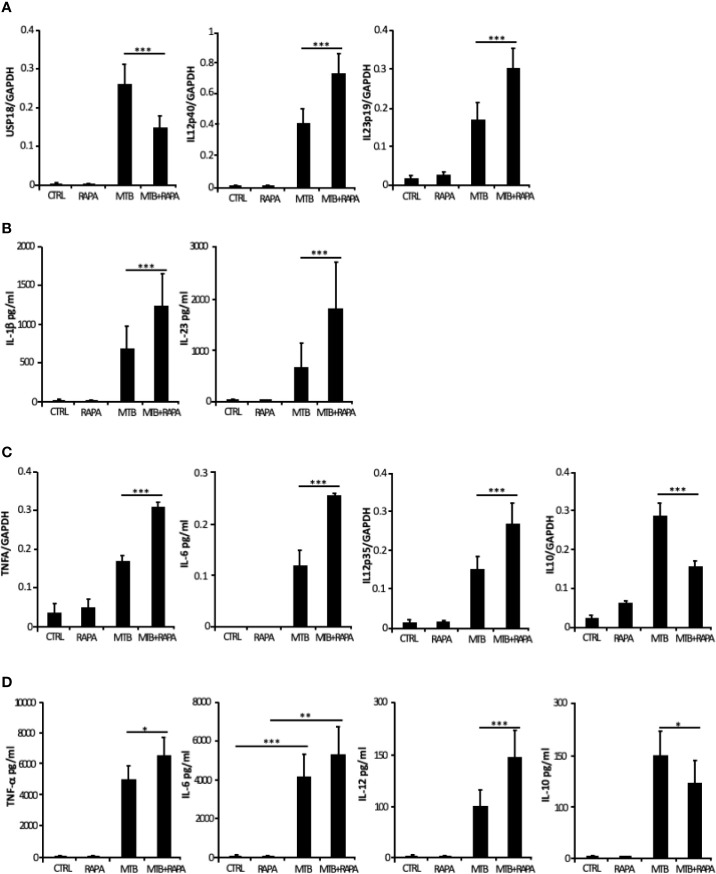
Validation of transcriptome data on cytokine expression and production. Total RNA was extracted from untreated DC (CTRL), DC stimulated for 12 h with rapamycin (RAPA) or infected for 16 h with Mtb alone (MTB) or in combination with rapamycin added 4 h post infection (MTB+RAPA). For protein determination in culture supernatants, DC were left untreated (CTRL) or stimulated for 20 h with rapamycin (RAPA) or infected for 24 h with Mtb alone (MTB) or in combination with rapamycin added 4 h post infection (MTB+RAPA). **(A)** *USP18, IL12p40*, and *IL23p19* levels were evaluated by quantitative real time PCR. Data are represented as the mean copy number per sample ± standard error of 4 experiments performed with RNAs derived from a set of experiments independent than those used in transcriptome/translatome studies and that yielded similar results. **(B)** IL-1β production was evaluated by Inflammatory Cytokine bead array kit, while IL-23 release was measured by ELISA. The results represent mean values ± standard error of 8 independent experiments. **(C)** *TNFA*, *IL6*, *IL12p35*, and *IL10* levels were analyzed as described in **(A)**. **(D)** The secretion of TNF-α, IL-6, IL-12, and IL-10 was quantified by using Inflammatory Cytokine bead array kit. The results represent mean values ± standard error of 8 independent experiments. Significance was calculated by analysis of variance (ANOVA) followed by multiple comparison performed with Tukey’s test as specified in *Materials and Methods* section.

From the *in silico* analysis of transcriptome data, *IL-12B* (also known as *IL12p40*), *IL-23A* (also known as *IL23p19*), *IL-1A*, *IL-1B*, *IL-8*, *IRAK2*, *RIPK2* and *MAP3K8* were the top genes induced by Mtb and further stimulated by rapamycin. In addition, *USP18* - a key negative regulator of type I IFN signaling (21) - was found among genes transcriptionally down-regulated by rapamycin in Mtb-infected DC (**Table 2**). Validation by qRT-PCR was obtained for *USP18* and for *IL12p40* and *IL23p19* (encoding the IL-23 subunits) (**Figure 5A**).

**Figure 6:**
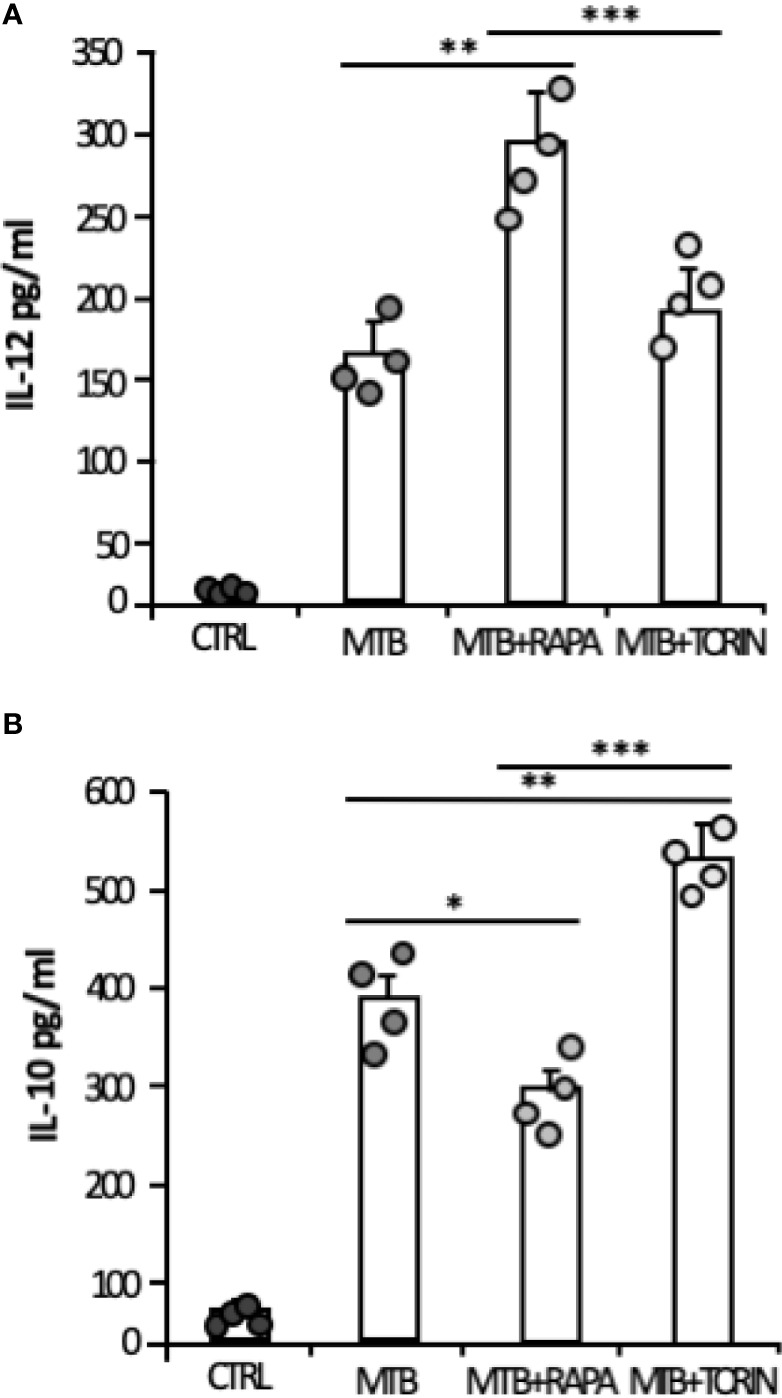
Effect of mTORC1 or mTORC1/mTORC2 inhibition on IL-12 and IL-10 cytokine production in Mtb-infected DC. DC were left untreated (CTRL) or stimulated for 20 h with rapamycin (RAPA) or infected for 24 h with Mtb (MTB) and treated or not with rapamycin (MTB+RAPA, added 4 h post infection) or with Torin1 (MTB+TORIN, added 4 h after infection). Secreted IL-12 **(A)** and IL-10 **(B)** were quantified by using Inflammatory Cytokine bead array kit. The results represent mean values ± standard error of 4 independent experiments. Circles represents values obtained, for the specified cytokine, from each single donor/experiment. Significance was calculated by analysis of variance (ANOVA) followed by multiple comparison performed with Tukey’s test as specified in *Materials and Methods* section.

Consistent with the gene expression results, the release of IL-1β and IL-23 was robustly increased when rapamycin was added to Mtb-infected DC (**Figure 5B**). Similarly, TNF-α and IL-6 mRNAs were up-regulated upon Mtb infection and further enhanced by rapamycin. Yet, rapamycin significantly promoted TNF-α release, while IL-6 production was not significantly regulated. As expected (9), the expression of *IL12p35* subunit and the secretion of IL-12 doubled following rapamycin addition to infected cells (**Figures 5C, D**). To better define the involvement of mTOR in cytokine regulation during Mtb infection, Torin1, an inhibitor of both mTORC1 and mTORC2, was used. The secretion of IL-12 was not significantly altered when Torin1 was added to infected cells (**Figure 6A**). Interestingly, the Mtb-induced expression of the anti-inflammatory cytokine IL-10 was reduced by rapamycin and enhanced by Torin1 (**Figures 5C, D** and **Figure 6B**). All together these data indicate that Mtb mainly impinges on the balance of pro- and anti-inflammatory cytokines *via* mTORC1 signaling.

### GSK-3β Behaves as Molecular Switch of Rapamycin-Driven Cytokine Expression During Mtb Infection

**Figure 7:**
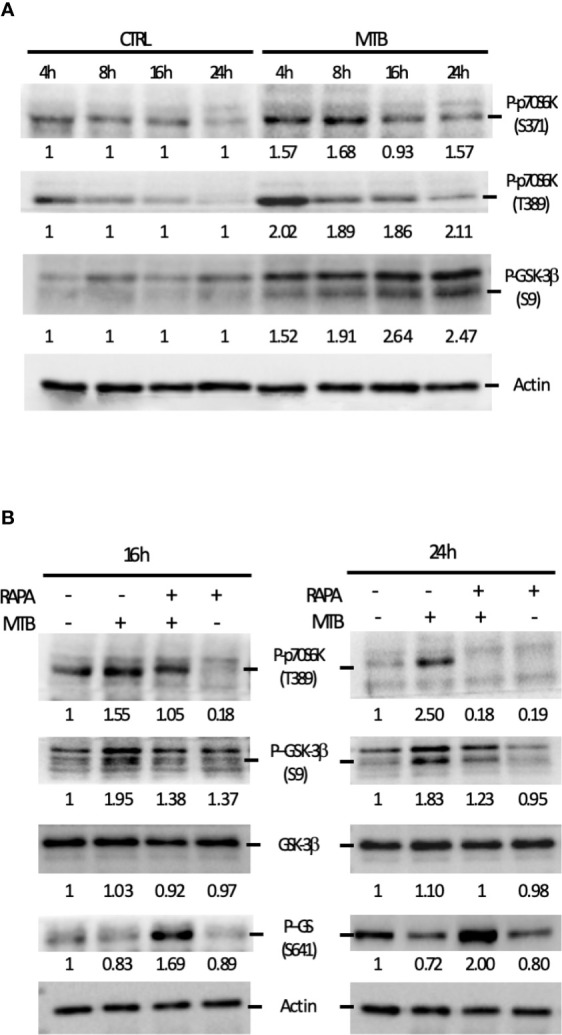
Analysis of the signaling cascades modulated by Mtb alone or in combination with rapamycin and leading to cytokine production. **(A)** DC were left untreated (CTRL) or infected with Mtb (MTB) in kinetic. Activation of p70S6K and GSK-3β was investigated by western blotting on whole cell extracts. Quantification of the phospho-p70S6K (S371 and T389) and phospho-GSK-3β (S9) bands is shown below each immunoblot. **(B)** The impact of rapamycin on p70S6K and GSK-3β was investigated at the indicated time points in DC left unstimulated (CTRL) or infected with Mtb alone (MTB) or in presence of rapamycin added 4 h after infection (MTB+RAPA). Quantification of phospho-p70S6K (S371 and T389), total GSK-3β, phospho-GSK-3β (S9) and phospho-GS (S641) is shown below each immunoblot. Actin levels were analyzed to verify protein content. A representative experiment out of 3 experiments that yielded similar results is shown.

Next, we performed a kinetic study to monitor the activation status of the 70 kDa ribosomal S6 kinase 1 (p70S6K), a Ser/Thr kinase and critical effector of the mTOR and the phosphoinositide-3 kinase (PI3K) signaling cascades (**Figure 7**), whose activation requires a multi-site phosphorylation (22, 23). We measured the level of phosphorylation at S371 and T389. While the level of phosphorylation at S371 was only slightly modulated by Mtb, a two-fold induction in p70S6K-T389 phosphorylation was observed at 4 h post-infection and lasted at least 24 h (**Figure 7A**). The time-dependent reduction of p70S6K-T389 phosphorylation in control cells was likely due to deprivation of GM-CSF (24) before the infection.

These data prompted us to investigate whether the fine modulation of pro- and anti-inflammatory cytokine levels in our experimental model involved GSK-3β, a constitutively active Ser/Thr kinase, which, when phosphorylated at the inhibitory S9 site by p70S6K, undergoes proteasomal degradation (25–27). To address this hypothesis, GSK-3β phosphorylation status was investigated. A gradual increase in the phosphorylation level at S9 site was revealed indicating that, in DC, GSK-3β is rapidly and persistently turned off upon Mtb infection (**Figure 7A**). Interestingly, phosphorylation of p70S6K at T389 was lowered by the addition of rapamycin in both control and Mtb-infected DC at 16 and 24 h, with a more pronounced effect in infected cells at the latter time point (**Figure 7B**). Similarly, GSK-3β phosphorylation at S9 site was abrogated by rapamycin, which in turn promoted also the phosphorylation of glycogen synthase (GS), one of the canonical substrates of GSK-3β. All together, these results indicate that rapamycin prevents the inhibition of GSK-3β.

Next, we studied whether the activation of the mitogen-activated protein kinases (MAPK) p44/42 and p38 - well-known regulators of the pro- and anti-inflammatory cytokines during Mtb infection (11, 28, 29) - was influenced by rapamycin in Mtb-infected DC. A kinetic analysis was performed to monitor the phosphorylation/activation status of these proteins (**Supplementary Figure 4**). An increase in p38 phosphorylation at T180/Y182 was observed as early as 4 h post-infection, while p44/42 activation was unchanged. Rapamycin weakly increased p38 phosphorylation in both control and Mtb-infected DC (**Supplementary Figures 4A, B**).

**Figure 8:**
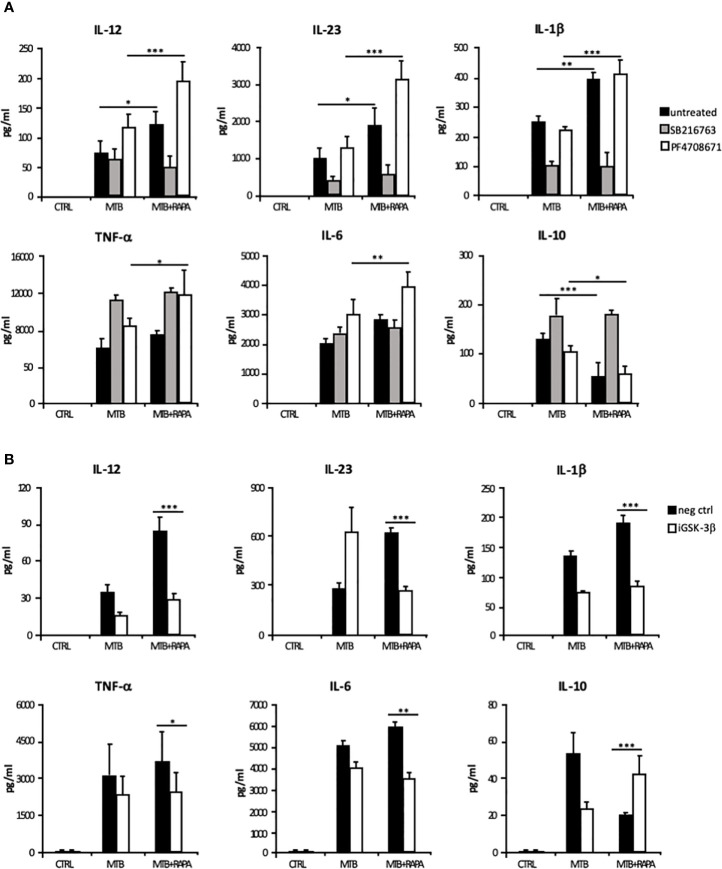
Characterization of GSK-3β and p70S6K involvement in Mtb-mediated cytokine production during rapamycin treatment of human DC. **(A)** DC were left unstimulated (CTRL) or infected for 24 h with Mtb alone (MTB) or in combination with rapamycin added 4 h post infection (MTB+RAPA), with or without the GSK-3β (SB216763) or the p70S6K (PF4708671) inhibitors. IL-12, IL-1β, TNF-α, IL-6 and IL-10 production was evaluated by Inflammatory Cytokine bead array kit, while IL-23 release was measured by ELISA. **(B)** DC were transfected with 100 nM of siRNA specific for GSK-3β (iGSK-3β) or with a control siRNA (neg ctrl) for 8 h and then left unstimulated (CTRL) or infected for 24 h with Mtb alone or in combination with rapamycin. Cytokine amount was determined as in **(A)**. The results represent mean values ± standard error of 4 independent experiments. Significance was calculated by analysis of variance (ANOVA) followed by multiple comparison performed with Tukey’s test as specified in *Materials and Methods* section.

The rapamycin effect on the p70S6K/GSK-3β axis was further investigated by analyzing the release of the pro-inflammatory/regulatory cytokines in the presence of a GSK-3β chemical inhibitor, SB216763. In this experimental setting, rapamycin lost its impact on cytokine secretion. In contrast, the inhibition of p70S6K by PF4708671 cooperated with rapamycin in enhancing pro-inflammatory cytokine expression in Mtb-infected DC (**Figure 8A** and **Supplementary Figure 5A**).

The inhibition of p38 signaling by SB203580 or SB202129 also mitigated the production of IL-12 and IL-10 induced by Mtb alone or in presence of rapamycin. On the other hand, p44/42 inhibition by PD980509 did not affect IL-12 production, but it reduced IL-10 secretion by Mtb-infected DC irrespective of the presence of rapamycin (**Supplementary Figure 4C**).

The involvement of GSK-3β in the rapamycin effect on cytokine expression was further analyzed by silencing GSK-3β in Mtb-infected DC with a specific siRNA (iGSK-3β) (**Figure 8B** and **Supplementary Figure 5B**). DC were also transfected with a non-specific siRNA as negative control (neg ctrl). Upon GSK-3β silencing, the levels of IL-12, IL-23, IL1β, TNF-α and IL-6 were not further enhanced by rapamycin, while the level of IL-10 was increased. As observed for the chemical inhibition (**Supplementary Figure 5A**), GSK-3β silencing impacted also on *IL-12* and *IL-10* mRNA levels (**Supplementary Figure 5B**). Taken together, these results support the view that in Mtb-infected DC rapamycin modulates pro- and anti-inflammatory cytokine production through the regulation of mTOR/GSK-3β axis.

## Discussion

For several decades major efforts in TB treatment have focused on the development of antibiotics targeting Mtb. The current regimens for drug-susceptible TB requires the administration of a cocktail of antibiotics for at least 6 months, thus making the cure complicated to administer, lengthy, hepatotoxic and not well tolerated by the patients. These drawbacks lead to poor adherence to the cure, low success rates and, consequently, high risk of development of drug-resistant TB (2, 30). Indeed, recently, the burden of TB disease has been exacerbated by the emergence of rifampicin-resistant, multidrug-resistant and extensively-drug resistant Mtb strains whose treatment requires the administration of second-line drugs for at least 9 months and even up to 20 months (1). In this scenario, the host-directed therapies (HDT) have garnered international interest, moving from the concept that the modulation of host defense contributes to the control and resolution of the infection, overcoming the problem of acquired resistance to pathogen-directed therapies (31). Moreover, HDT can control the antimicrobial resistance by either boosting host-cellular responses or activating innate and adaptive immunity and, thus, immunological memory against the pathogens (32). Therefore, the development of HDT is particularly useful for diseases like TB, where the host immune response can successfully limit infection in the majority of latently infected individuals. In this context, there is growing evidence that HDT, such as those based on autophagy enhancement, could be successfully employed as therapeutics for Mtb treatment (10).

Among autophagy inducers, rapamycin and its analogs (rapalogs) have been shown to possess interesting immunoregulatory properties and their use is of interest to the field (33). However, the precise molecular mechanism by which rapamycin exerts its function has not been fully elucidated. In line with evidences demonstrating a multi-level regulation of immune cell activity, including RNA transcription and stability as well as translation (34, 35), the transcriptome and the translatome profiles were obtained to study in depth the effect of rapamycin in human primary DC infected with Mtb. Our data show for the first time that rapamycin selectively tunes cytokine production in Mtb-infected DC by two mechanisms: i) promoting the association of type I and III IFN mRNAs to polysome chains and, in turn, their translation; ii) preventing the inhibition of GSK-3β to tune pro- and anti-inflammatory cytokine production. In this scenario, mTOR plays a central role in the transcriptional and translational regulation of immune cell function (34, 35). Indeed, mTOR regulates p70S6K1 phosphorylation, which in turn blocks the activity of the eukaryotic translation initiation factor 4E-binding protein 1 (4E-BP1) and promotes mRNA translation. Yet, the contribution of mTOR to phagocytic cell functions during Mtb infection is poorly defined (36, 37). Our translatome analysis of Mtb-infected DC identified, among the rapamycin-induced mRNAs efficiently loaded on polysome, *IFNB1* and *IFNL1*, as well as three ISG - *IFIH1*, *IFIT3* and *IRF7* - whose role in Mtb infection was recently described (18, 20, 38).

Interestingly, in addition to activating host DNA sensors, like cyclic GMP-AMP synthase and stimulator of IFN genes (39, 40), Mtb RNA may trigger also through RNA sensing molecules (18, 20, 41, 42). Moreover, few studies reported that Mtb RNA is present in endosome-derived membrane vesicles, is released into macrophage cytosol by means of SecA and ESX-1 secretion systems and stimulates IFN-β production through of retinoic acid-inducible gene (RIG-I), IFIH1, MAVS, protein kinase R (PKR) and IRF7 signaling cascades (18, 20). In this context, we found that the level of *USP18* - encoding a factor limiting long-lasting IFN response (21, 43) - was reduced by rapamycin in Mtb-infected DC, further suggesting a link between IFN signaling activation and mTOR inhibition. Collectively, the enhanced polysome loading of the above mentioned ISGs, combined with the reduced expression of *USP18*, suggested that mTORC1 inhibition by rapamycin promotes an IFN signature in Mtb-infected DC. Thus, in addition to the well-known effect of PI3K-mTOR pathway on IFN signature (44, 45), our data suggest a detrimental mTORC1 effect on ISG induction, and likely a beneficial mTORC2 activity as shown in previous studies (46, 47).

**Figure 9:**
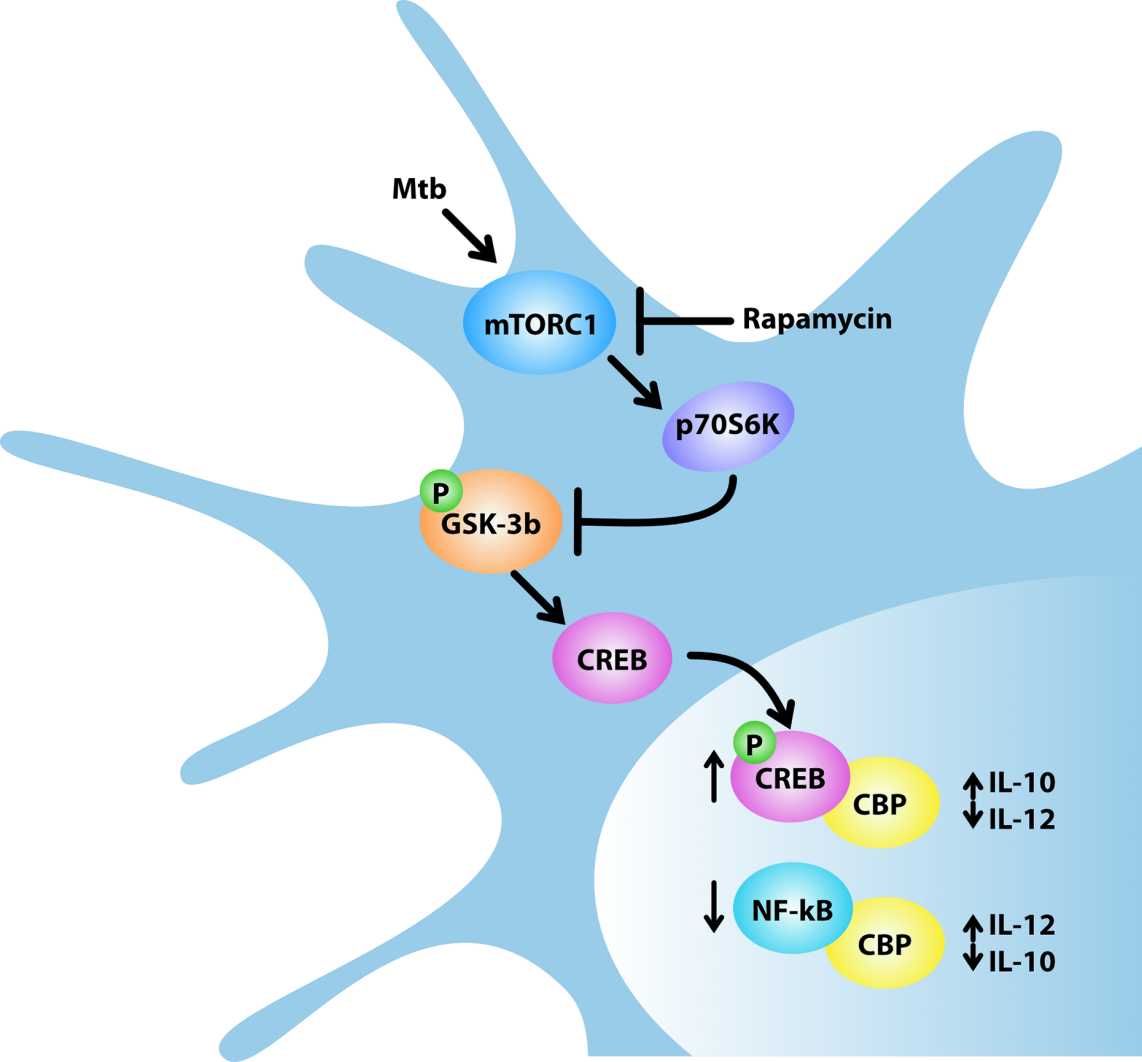
Model for rapamycin-triggered cytokine regulation during Mtb infection. Mtb infection of human DC activates mTORC1/p70S6K axis that phosphorylates and, in turn, inhibits the multifunctional protein kinase GSK-3β. The mTORC1/p70S6K-mediated suppression of GSK-3β favors the nuclear translocation of CREB and in turn its binding to the coactivator of transcription CBP, while it reduces the amount of NF-kB p65 associated with CBP. In Mtb-infected DC, rapamycin, by blocking mTORC1, prevents GSK-3β inhibition thus impinging on IL-12/IL-10 expression and production.

The unexpected role of mTOR in the control of cytokine production in human DC infected with Mtb was further analyzed through a global transcriptome. This study showed that rapamycin likely promotes transcription of a panel of pro-inflammatory/regulatory cytokines including IL-23, TNF-α, IL-1β and IL-6 in addition to IL-12 and inhibits the expression of the anti-inflammatory IL-10. These findings mirror previous data on rapamycin-driven CD4^+^ T-cell activation and differentiation *via* IL-12/IL10 release from LPS-stimulated DC (48). Our data were validated by measuring the level of secreted cytokines. In addition to the PI3K/mTOR pathway, it has been shown that the constitutively active kinase GSK-3β contributes to the balance of pro- and anti-inflammatory cytokines in LPS- or *Listeria monocytogenes*-stimulated APC (25, 37, 49, 50). In particular, the mTOR-dependent inhibition of GSK-3β favors nuclear translocation of CREB and in turn its binding to the coactivator of transcription CBP, while it reduces the amount of NF-kB p65 associated with CBP, thus impacting on the balance of pro- and anti-inflammatory cytokines (25, 26). In line with these evidences, our results indicate that: i) Mtb inactivates GSK-3β by acting on mTOR/p70S6K; ii) rapamycin overturns this block by suppressing the inhibitory phosphorylation of GSK-3β; iii) in Mtb-infected DC, rapamycin promotes the release of pro-inflammatory/regulatory cytokines while it reduces the secretion of IL-10 (**Figure 9**). The key function of GSK-3β was confirmed by lower levels of secreted IL-12, IL-23 and IL-1β and higher level of IL-10 following silencing or pharmacological inhibition of GSK-3β in Mtb-infected DC.

Given the relevance of the IL-12/IL-23 axis in driving the differentiation of CD4^+^ T-cells towards a protective Th1 phenotype (9, 51–53), our data suggest that GSK-3β may be a potential target for the development of novel HDT to fight Mtb infection. This possibility is relevant also in light of the rapamycin-driven reduction of IL-10, a cytokine detrimental in TB, acting on the stability of TB granuloma, on Mtb persistence into the host,and promoting Mtb evasion from the autophagic machinery (54–56). Consistent with these observations, the use of inhalable particles containing rapamycin in combination with anti-TB drugs may contribute to lung tissue regeneration and, at the same time, to Mtb killing (57). Accordingly, the combined analysis of DC transcriptome and translatome corroborates the exploitation of molecules, such as rapamycin or GSK-3β modulators, as novel HDT to treat TB. Finally, these findings might be relevant for other respiratory infections, including those caused by coronaviruses, where the aerosol delivery of molecules tuning autophagy and mTOR/GSK-3β axis might exert a therapeutic effect.

## Data Availability Statement

The data sets presented in this study can be found in online repositories. The names of the repository/repositories and accession number(s) can be found in the article/supplementary material.

## Author Contributions

ME participated in experimental design, performed experiments, analyzed data, and prepared the manuscript. MS and VL performed experiments and analyzed data. MP, MCr, FR, EG, GM, OP, and RS performed experiments. ML discussed the data. MCa and RN contributed to data interpretation. SP contributed to data interpretation and manuscript writing. EC participated in experimental design, data analysis, and manuscript writing. All authors contributed to the article and approved the submitted version.

## Funding

This work was supported by grant RF-2010-235199 (to EC) from Italian Ministry of Health and grant FILAS-RU-2014-1036 (to EC) from Lazio Region. The funders had no role in study design, data collection and analysis, decision to publish, or preparation of the manuscript.

## Conflict of Interest

The authors declare that the research was conducted in the absence of any commercial or financial relationships that could be construed as a potential conflict of interest.​​​​​​​​​​​​​​​​​​​​
