# Supplementary material for: Genome-Wide Gene Expression Analysis of Mtb-Infected DC Highlights the Rapamycin-Driven Modulation of Regulatory Cytokines via the mTOR/GSK-3β Axis
Source: Front Immunol. 2021 Apr 16;12:649475. doi: 10.3389/fimmu.2021.649475 (PMC8086600; doi:10.3389/fimmu.2021.649475)
Supplement: Supplementary file 1 [file Image_1.pdf]

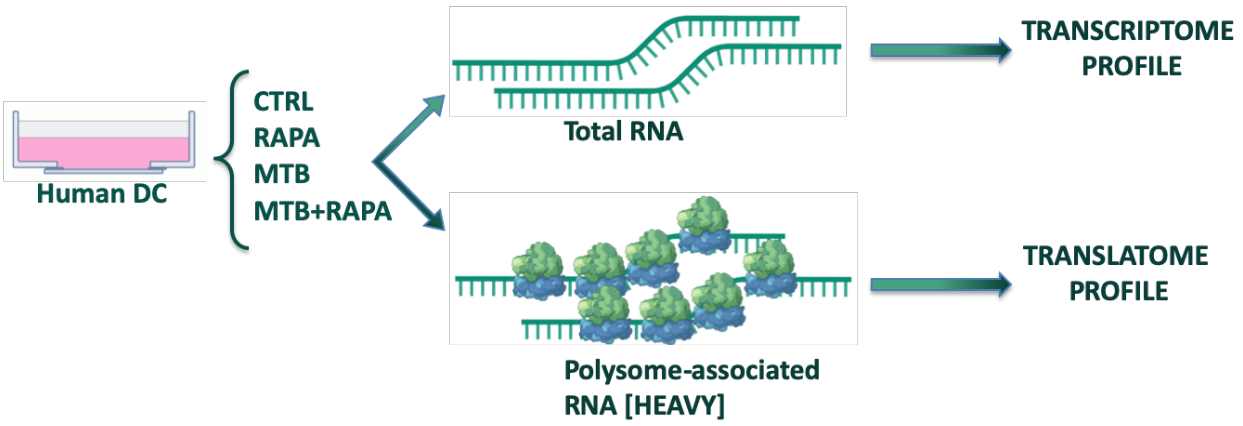

Supplementary Figure 1. Schematic representation of the experimental design of the study. Human DC prepared from 4 healthy donors were either left untreated (CTRL) or stimulated with rapamycin (RAPA) or infected with Mtb alone (MTB) or in combination with rapamycin added 4 hours after infection (MTB+RAPA) for 16 hours. Total and polysome-associated RNA were isolated and then used to generate transcriptome and translatome profiles respectively.

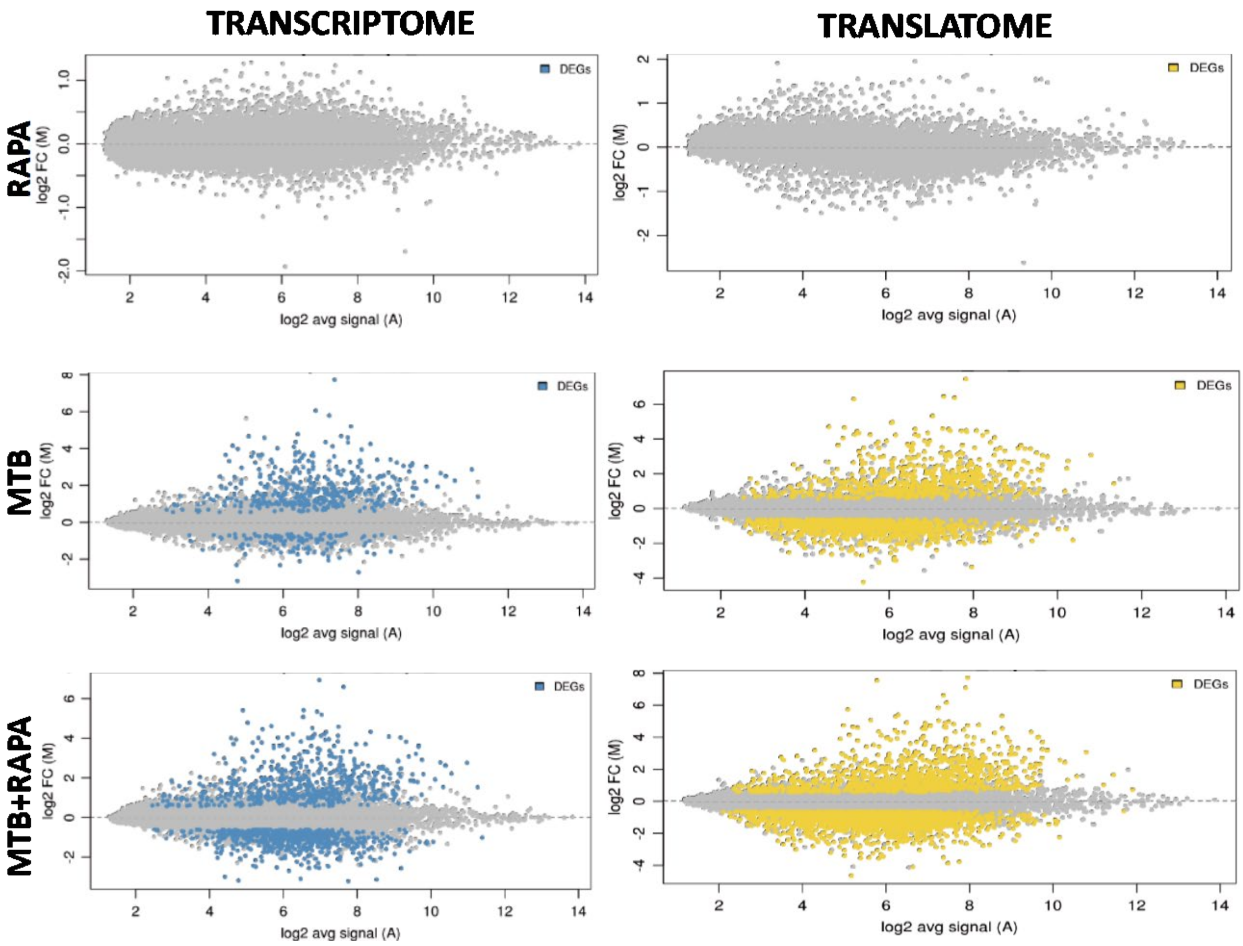

Supplementary Figure 2. Differentially expressed genes modulated in transcriptome and translatome of human DC infected with Mtb and treated with the autophagy inducer rapamycin.

MA-plots representing the logged intensity ratio (M) versus the mean logged intensities (A) of genes significantly modulated in human DC treated with rapamycin (RAPA) and/or infected with Mtb (MTB) vs uninfected cells (adjusted p-value [FDR] < 0.01 and at least 1.5 log2 fold change). Colored points represent significant genes de-regulated in transcriptome (blue) and in translatome (yellow).

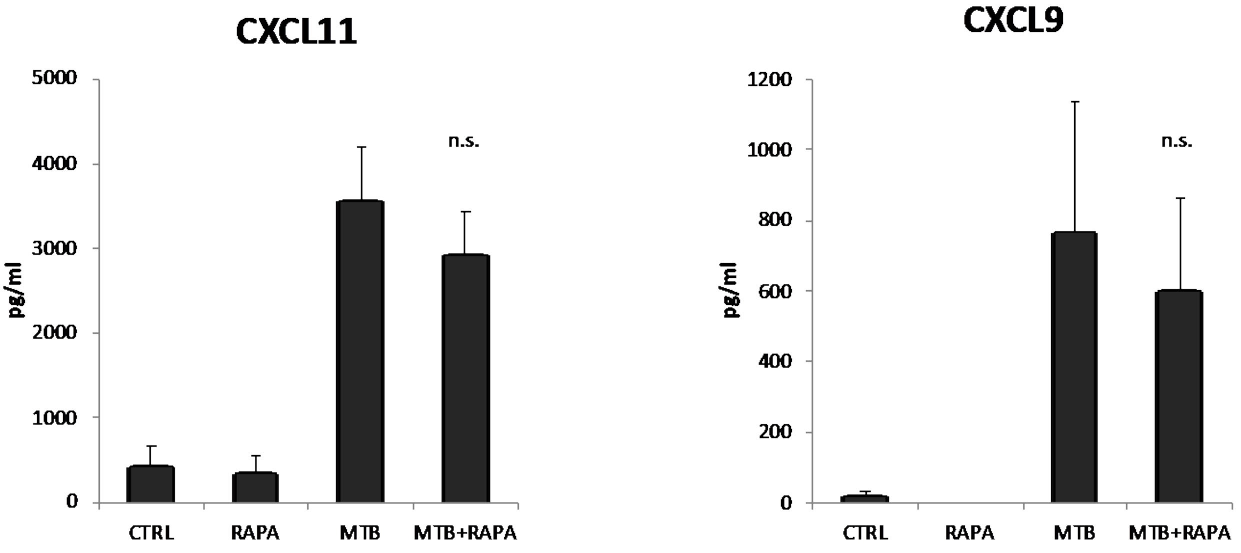

Supplementary Figure 3. Effect of Mtb infection and rapamycin treatment on CXCL9 and CXCL11 secretion in DC.

DC were left untreated (CTRL) or stimulated for 20 hours with rapamycin (RAPA) or infected for 24 hours with Mtb (MTB) and treated or not with rapamycin (MTB+RAPA, added 4 hours after infection). CXCL9 and CXCL11 release was measured by specific ELISA kits. The results represent mean values ± standard error of 4 independent experiments.

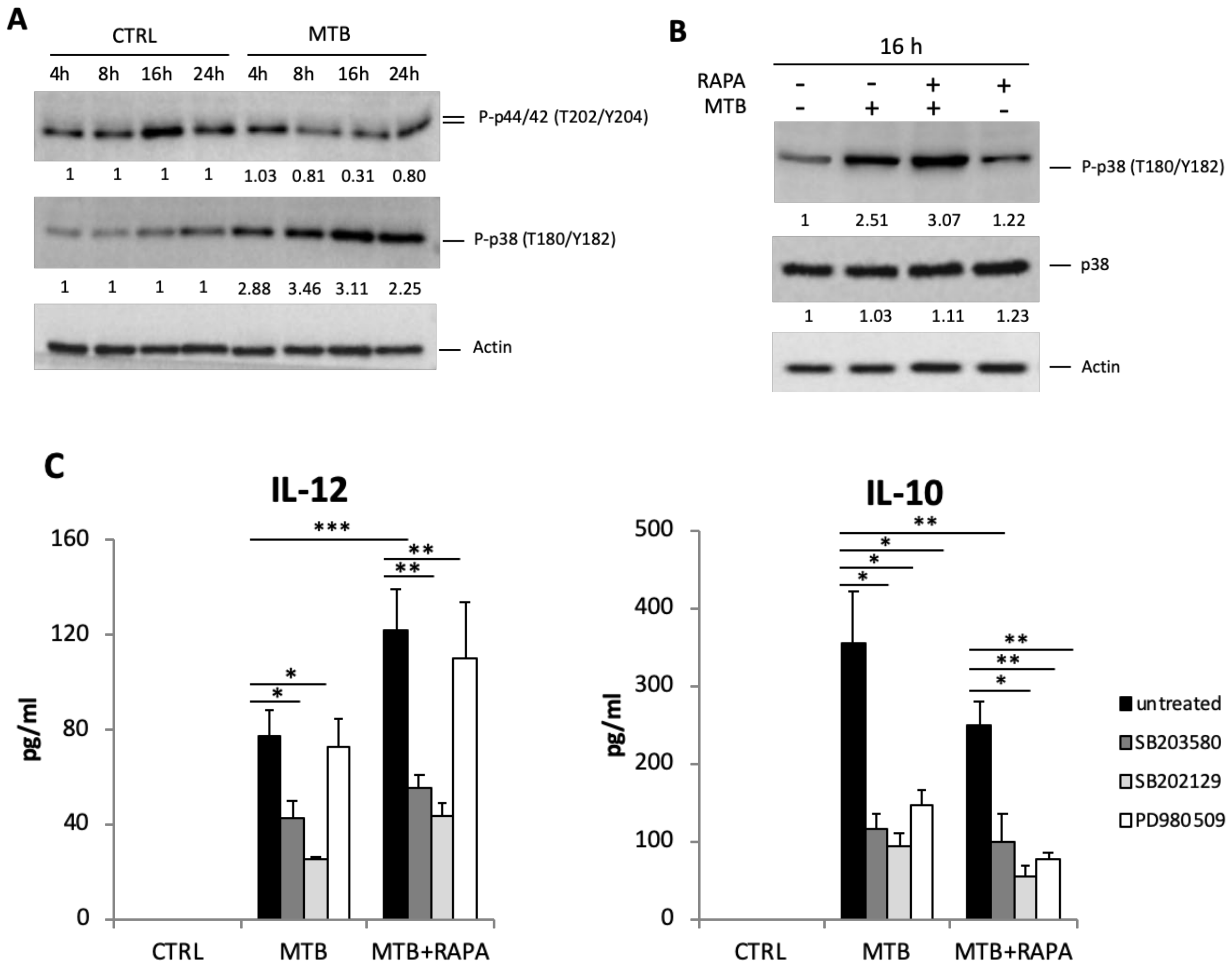

Supplementary Figure 4. Effect of p44/42 or p38 inhibition on the capacity of rapamycin to modulate the levels of IL-12 and IL-10 during Mtb-infection.

(**A**) DC were left untreated (CTRL) or infected with Mtb (MTB) in kinetic. The activation of p44/42 and p38 was investigated by western blotting on whole cell extracts using specific antibodies. Band intensity quantification for P-p44/42 (T2020/Y204) and P-p38 (T180/Y182) and total p38 is indicated below the blot. Actin levels were analyzed to verify protein loading. A representative experiment out of 3 experiments that yielded similar results is shown. (**B**) Impact of rapamycin on p38 activation was investigated by western blotting in DC left unstimulated (CTRL) or infected with Mtb (MTB) in presence or absence of rapamycin (MTB+RAPA, added 4 hours after infection) at the indicated time points. Band intensity quantification was performed as described in A. A representative experiment out of 3 experiments that yielded similar results is shown. (**C**) DC were left unstimulated (CTRL) or infected for 24 hours with Mtb (MTB) alone or in combination with rapamycin (MTB+RAPA, added 4 hours after infection) and with or without the p38 (SB203580 or SB202129) or the p44/42 inhibitor (PD980509). IL-12 and IL-10 production was evaluated by Inflammatory Cytokine kit. The results represent mean values ± standard error of 4 independent experiments. Significance was calculated by analysis of variance (ANOVA) followed by multiple comparison performed with Tukey's test as specified in material and method section.

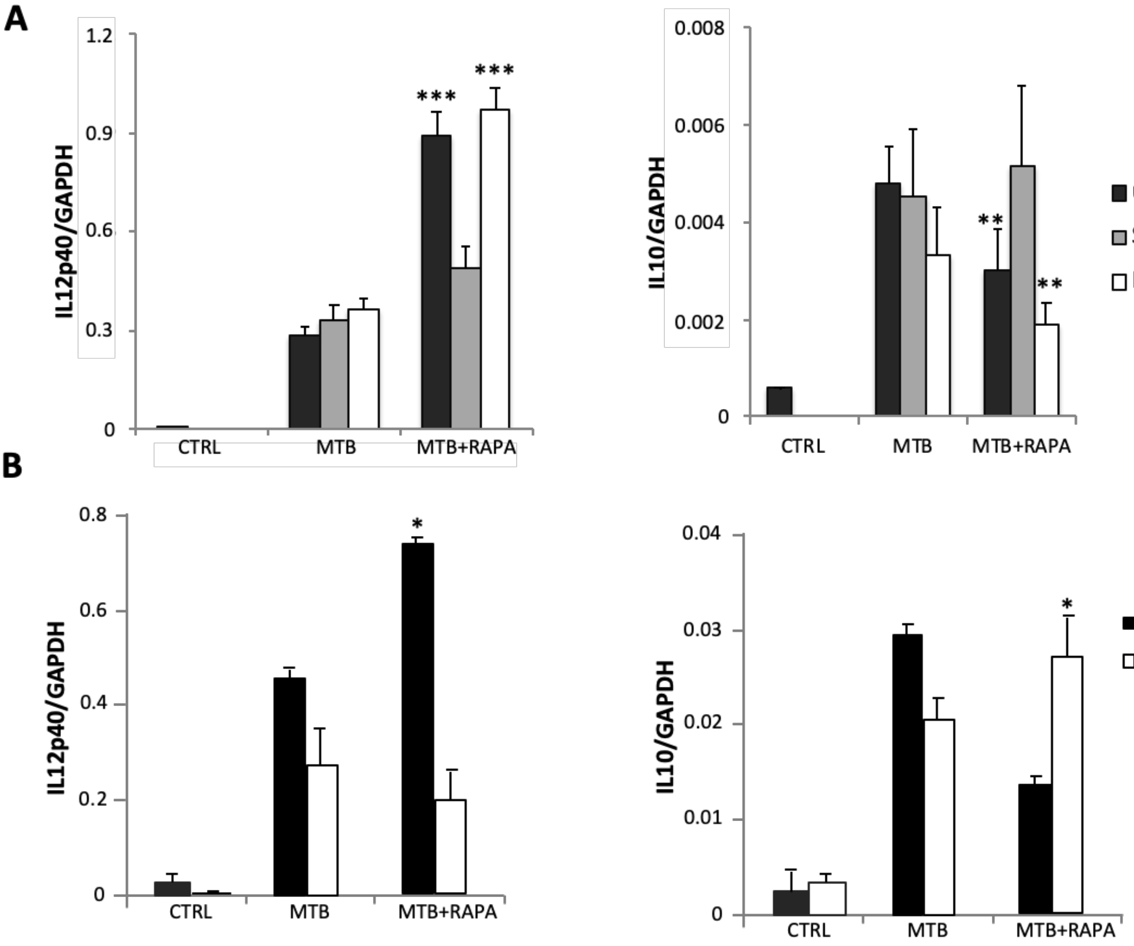

Supplementary Figure 5. Effect of inhibition of GSK-3β and p70S6K and of GSK-3β silencing on the capacity of rapamycin to modulate IL-12p40 and IL-10 expression during Mtb-infection. (**A**) Total RNA was extracted from untreated DC (CTRL), DC stimulated with rapamycin for 20 hours (RAPA) or DC infected for 24 hours with Mtb (MTB) and treated or not with rapamycin (MTB+RAPA, added 4 hours after infection) and with or without the GSK-3β (SB216763) or the p70S6K inhibitors (PF4708671). Real-time PCR was conducted to evaluate the expression of IL-12p40 and IL-10. (**B**) DC were left unstimulated (CTRL) or infected for 8 hours with Mtb at MOI of 1 bacterium/cell alone or in combination with rapamycin and then transfected with 100 nM of siRNA specific for GSK-3β (iGSK3b) or with a negative control siRNA (neg ctrl) for 24 hours. The expression of IL-12p40 and IL-10 was analyzed by real-time PCR on extracted total RNA. Data were normalized to GAPDH using the equation $2^{-\Delta Ct}$. The results shown were mean of 4 experiments performed with RNAs derived from independent experiments. Significance was calculated by the analysis of variance (ANOVA) followed by multiple comparison performed with Tukey's test (see Materials and Methods).
